# Embolization for massive bleeding due to spontaneous left external iliac vein rupture: report of a successful case

**DOI:** 10.1186/s42155-021-00219-5

**Published:** 2021-04-01

**Authors:** Eijun Sueyoshi, Ichiro Sakamoto, Masataka Uetani

**Affiliations:** Department of Radiological Sciences, Graduate School of Biomedical Sciences, Nagasaki University, 1-7-1 Sakamoto, Nagasaki, 852-8501 Japan

**Keywords:** Computed tomography, Catheter interventions, Embolization, Endovascular therapy

## Abstract

**Background:**

Spontaneous rupture of the iliac vein has rarely been reported. Its associated hypovolemic shock-related symptoms and signs, including syncope and hypotension, have been observed in most of these cases. Successful transcatheter venous embolization for massive bleeding due to spontaneous rupture of the external iliac vein was herein reported.

**Case presentation:**

An 82-year-old female patient developed sudden left lower abdominal and back pain. Immediately, she lost consciousness and went into shock. CT images of her abdomen revealed a huge retroperitoneal hematoma, with leakage of contrast medium in the hematoma in the left flank. These findings suggested left external iliac vein rupture.

Open surgery was considered; however, since the patient’s condition may have deteriorated further due to the time needed to prepare for surgery, including general anesthesia, transcatheter venous embolization of the left iliac vein was selected. A 5.2-Fr compliant balloon catheter (nominal diameter of 10 mm) was inflated at the distal site of the external iliac vein to reduce extravasation. N-butyl-2-cyanoacrylate (NBCA) was mixed with Lipiodol at a ratio of 1:2. The left Iliac vein was filled and completely embolized with the NBCA/Lipiodol mixture (total injected volume, 5 mL) using a 1.8-Fr microcatheter. After embolization, the patient quickly. An inferior vena cava filter was placed 1 day after embolization.

**Conclusion:**

Spontaneous rupture of the iliac vein is a very rare and lethal condition. Transcatheter venous embolization may control potentially life-threatening bleeding. Rapid bleeding control in a critical condition is facilitated by this minimally invasive approach.

## Background

External iliac vein rupture is very rare, but it necessitates emergent treatment (Jiang et al. 2010; Kim IH et al. 2011). This lethal condition was provoked by the bleed caused hypovolemic shock related symptoms and signs, including syncope and hypotension in most cases. In the emergency room, this condition has been mistaken for traumatic or gynecological emergency surgical cases because of the accompanying abdominal distension and syncope-related trauma. The findings of leaked contrast media suggesting vessel rupture were not easily found in abdominopelvic CT on account of heavily compressed hematoma (Kim IH et al. 2011). Immediate resuscitation is imperative, but the appropriate management was disturbed in some cases (Jiang et al. 2010; Kim IH et al. 2011).

We herein report a case of successful transcatheter embolization for massive bleeding due to spontaneous rupture of the external iliac vein. We also present imaging findings. To the best of our knowledge, this is the first case report to show successful embolization for spontaneous rupture of the external iliac vein.

## Case presentation

An 82-year-old female patient with left knee pain and left leg edema was admitted to the emergency room. She had a history of hypertension and 1 week of constipation, with no other diseases or history of trauma. Shortly thereafter, she developed sudden left lower abdominal and back pain. She lost consciousness and went into shock. Blood pressure was 50/36 mmHg and her pulse rate was 150 beats per minute. The results of a complete blood count showed anemia, with a hemoglobin concentration of 7.1 g/dL and platelet count of 84,000/μL, and no other abnormal data.

**Fig. 1:**
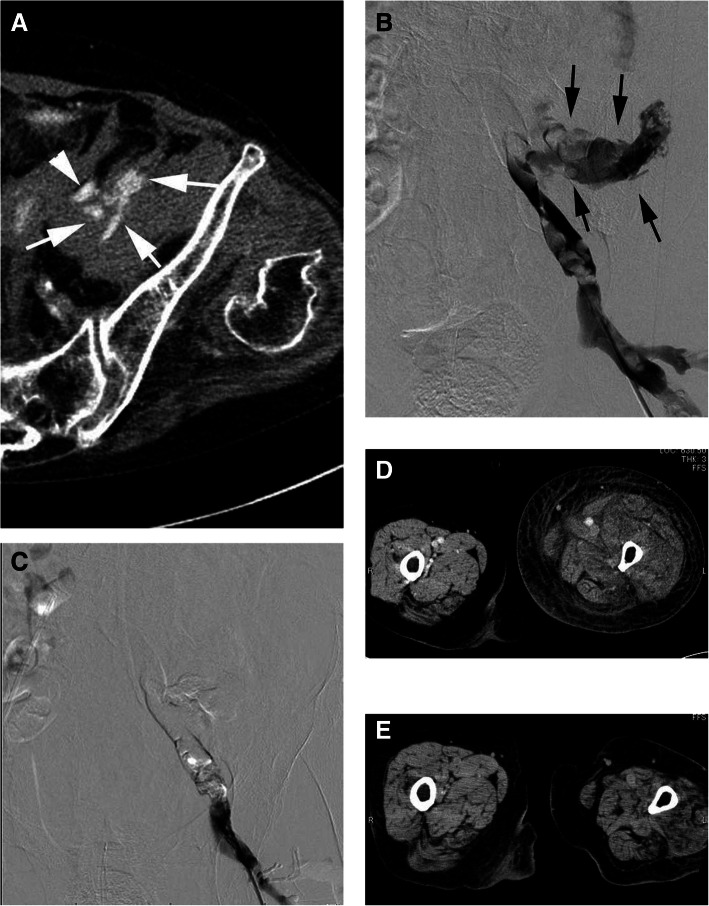
**a**. Contrast-enhanced CT of the venous phase shows extravasation (arrows) from the lt. external iliac vein (arrowhead). **b**. DSA shows marked extravasation from the laceration to the adjacent site of the left internal iliac vein (arrows). A thrombus occluded the left common iliac vein. **c**. DSA shows that the left iliac vein hwas filled and fully embolized with the NBCA/Lipiodol mixture. **d**. Two days after following embolization, CT shows left leg swelling similar to deep venous thrombosis. **e**. Six months after embolization, CT shows the resolution of left leg swelling

Non-contrast and contrast-enhanced CT was performed. CT images of the abdomen revealed a large retroperitoneal hematoma, with the leakage of contrast medium in the hematoma into the left flank. The left common iliac vein was dilated with a thrombus, and the origin of the bleeding was apparent (Fig. 1a). These findings suggested left external iliac vein rupture.

Open surgery was considered,; however, since the patient’s condition may have deteriorated further due to the time needed to prepare for surgery, including general anesthesia, transcatheter venous embolization of the left iliac vein was selected.

The procedure was performed under local anesthesia. Sheaths (5-Fr) were introduced at the left common femoral vein. Digital subtraction angiography (DSA) from the left external iliac vein by using a 5.2-Fr compliant balloon catheter (nominal diameter of 10 mm) (Selecon MP Catheter II, Terumo Clinical Supply Co., Ltd., Gifu, Japan) inserted from the sheath showed massive extravasation from the laceration to the adjoining site of the left internal iliac vein. The left common iliac vein was occluded by thrombus (Fig. 1b).

After the 5.2-Fr balloon catheter had been inflated at the distal site of the external iliac vein to reduce the extravasation. N-butyl-2-cyanoacrylate (NBCA) was mixed with Lipiodol (Guerbet, Villepinte, France) at a ratio of 1:2. The left Iliac vein was filled and completely embolized with the NBCA/Lipiodol mixture (total injected volume, 5 mL) using a 1.8-Fr microcatheter (Carnelian® PIXIE ER, Tokai Medical Products, Inc., Aichi, Japan) (Fig. 1c).

After embolization, the patient quickly recovered. One day after embolization, an inferior vena cava filter (FilterWire*,Boston Scientific, Massachusetts,USA)* was temporarily implanted to prevent pulmonary embolism due to the presence of a large amount of thrombus in the left iliac artery. One week later, the filter was removed. After embolization, this patient developed left leg swelling similar to deep vein thrombosis (Fig. 1d). We administered low molecular weight heparin, which was subsequently replaced with warfarin and applied compressive stockings. The patient was followed up 6 months later. Leg edema gradually resolved. Due to the development of collateral circulation and the amelioration of deep vein thrombosis, the administration of warfarin was stopped at 2 months (Fig. 1e).

Informed consent was obtained from the patient for the publication of this case report and any accompanying images.

## Discussion

Spontaneous rupture of the iliac vein has rarely been reported; only approximately 30 cases have been published to date (Jiang et al. 2010; Kim IH et al. 2011).

Despite several proposed etiologies, including venous hypertension and constipation, the underlying cause remains unknown (Jazayeri et al. 2002; Kwon et al. 2004).

The majority of cases have been complicated by deep venous thrombosis. Local venous hypertension due to deep venous thrombosis and venous wall weakness developing secondary to thrombophlebitis may be of etiological significance (DePass 1998; Plate G et al.^1995^). The present case had deep venous thrombosis and constipation.

The associated hypovolemic shock-related symptoms and signs, including syncope and hypotension, have been examined in the majority of these cases. Previous studies revealed that the goal of surgical management was to maintain the continuity of the ruptured iliac vein by direct suture or bypass reconstruction (Jiang et al. 2010; Kwon et al. 2004). onto the best of our knowledge, effective transcatheter embolization for marked bleeding due to spontaneous rupture of the external iliac vein has not yet been reported.

Two cases of the endovascular management of iliac vein rupture during percutaneous interventions for occlusive lesions were previously reported. In these cases, covered stents were employed (Adams MK et al. 2012). In one case, external iliac vein rupture was repaired with an endovascular stent and open laparotomy for abdominal decompression (Chen YC et al. 2018).

The treatment using covered stents may have been more favorable than transcatheter venous embolization because covered stents are able to preserve the venous flow in the iliac vein. However, the patient’s condition was severe due to active and massive bleeding, and there were no covered stents in the hospital..

Following embolization in the present case, venous flow was impaired due to occlusion of the left iliac vein, leading to leg swelling. In the 6 month follow-up, leg edema gradually resolved. Postoperative anticoagulation and the development of collateral circulation may play important roles in maintaining venous flow and recovery.

## Conclusion

Spontaneous rupture of the iliac vein is a very rare and lethal condition. Transcatheter venous embolization can control potentially life-threatening bleeding and may be an appropriate alternative to direct open repair. Rapid bleeding control in a critical condition is facilitated by this minimally invasive approach.

## Data Availability

The relevant data have been included in the manuscript. The datasets used and analyzed during the current study are available from the corresponding author upon reasonable request.

## Abbreviations

CT: Computed tomography
Fr: French Gauge
DSA: Digital subtraction angiography
NBCA: N-butyl-2-cyanoacrylate

## References

[CR1] Adams MK, Anaya-Ayala JE, Davies MG (2012). Endovascular management of iliac vein rupture during percutaneous interventions for occlusive lesions. Ann Vasc Surg.

[CR2] Chen YC, Huang CL, Huang JW (2018). Endovascular stent can be the treatment of choice for spontaneous iliac vein rupture: a case report. Vasc Endovasc Surg.

[CR3] DePass IE (1998). Spontaneous common iliac vein rupture: a case report. Can J Surg.

[CR4] Jazayeri S, Tatou E, Cheynel N, Becker F, Brenot R, David M (2002). A spontaneous rupture of the external iliac vein revealed as a phlegmasia cerulea dolens with acute lower limb ischemia: case report and review of the literature. J Vasc Surg.

[CR5] Jiang J, Ding X, Zhang G, Su Q, Wang Z, Hu S (2010). Spontaneous retroperitoneal hematoma associated with iliac vein rupture. J Vasc Surg.

[CR6] Kim IH, Chon GR, Jo YS, Park SB, Han SD (2011). Spontaneous left external iliac vein rupture. J Korean Surg Soc.

[CR7] Kwon TW, Yang SM, Kim DK, Kim GE (2004). Spontaneous rupture of the left external iliac vein. Yonsei Med J.

[CR8] Plate G, Qvarfordt P (1995). Idiopathic ruptureof the iliac vein: case report. Eur J Surg.

